# Impact of pre-appointment contact and short message service alerts in reducing ‘Did Not Attend’ (DNA) rate on rapid access new patient breast clinics: a DGH perspective

**DOI:** 10.1186/s12913-020-05627-2

**Published:** 2020-08-17

**Authors:** Pasupathy Kiruparan, Nanthesh Kiruparan, Debasish Debnath

**Affiliations:** Breast Unit, Blackpool Victoria Hospital, Blackpool, FY3 8NR UK

**Keywords:** Outpatients, Breast, Patient appointment, Patient non-attendance, Telephone, Text message, Short message service

## Abstract

**Background:**

Failure to attend the clinic without prior intimation, known as “Did Not Attend” (DNA) is a significant global issue. There have been no published studies attempting to reduce DNA rates in breast clinics. We aimed to assess the impact of contacting patients prior to clinic attendance and Short Message Service (SMS) reminder on DNA rates in rapid access new patient breast clinics, evaluate ‘Could Not Attend’ (CNA) rate, and explore any correlation between age, sex, clinic days and sessions.

**Methods:**

Initially, DNAs at the rapid access new patient breast clinic between 01/04/2018 and 31/03/2019 at a district general hospital in the North-West of England was assessed (Cycle 1). Changes were introduced in terms of contacting patients prior to offering appointments, followed by SMS reminders nearer the clinic dates. Subsequently, DNA was reassessed between 01/10/2019 and 31/03/2020 (Cycle 2).

**Results:**

Following implementation of changes, DNA rate reduced from 8.2 to 4.1% (*p* < 0.00001). CNA rates were 0.9% (Cycle 1) and 1.1% (Cycle 2) [*p* = 0.36]. Evening clinics had the lowest DNA rates throughout. DNA patients in cycle 2 were significantly older than those in cycle 1 (*p* = 0.002).

**Conclusions:**

Contacting patients prior to clinic appointments and sending SMS reminders helped reduce DNA rates significantly in rapid access new patient breast clinics. Scheduling clinic sessions with least DNA rates, such as evening clinics, should be contemplated. One should be cautious of mobile phone technology that conveys SMS, which can potentially disadvantage the older age group. This model could be considered across the board to improve DNA rates.

## Background

*“Spare a thought for that empty chair…”* [1]

Failure to attend outpatient appointments without any advance intimation, commonly known as “Did Not Attend” (DNA) in the UK, is a common problem encountered globally [2]. NHS Scotland launched the ‘Spare a thought for that empty chair…it could be costing more than you think’ campaign to raise awareness on DNA [1]. Such clinic DNAs delay patient health care management, increase waiting times and can impact on patient satisfaction [3]. The non-attendance of medical appointments is a multifactorial issue that negatively affects patient health, physician time, and resource management [4]. Rescheduling appointments stretches the already limited services even further.

Various studies have explored the underlying reasons for DNAs [5]. Issues such as transport, childcare, work commitments and forgetfulness are documented to be associated with DNAs [5, 6]. Factors noted to be predictive of DNA-behaviour include previous DNA history, high lead time, younger age and distance from the clinic [7].

According to National Health Service (NHS) England quarterly activity return data (2008–2018), the average DNA rate for first outpatient appointments has been 8.7% [8]. DNA rate has fallen in most quarters, despite an increase in both first and subsequent outpatient attendances since 2008/09 [9]. However, the DNA rate in recent quarters appears to have levelled off. For example, DNA rate for quarter 2 of 2017/2018 and 2018/2019 has been 8.9% [9]. The cost of missed outpatient clinic appointments in the NHS England in 2017/2018 was projected to be £1 billion [10]. This undoubtedly causes a huge economic burden on an already stretched NHS.

In contrast to DNA (also known as ‘No-Show’), a patient may be categorised as ‘Could Not Attend’ (CNA) when the hospital is notified in advance of patient’s unavailability to attend on the offered admission date, or for any appointment [11]. CNA rate can be quite high. For example, CNA rate for the NHS Northern Ireland was 11.3% for 2018/2019 [12].

Healthcare providers are increasingly using Short Message Service (SMS) or text message-based reminders to reduce DNA rates [13]. Some hospitals have tried to compensate DNAs by overbooking clinics, with ensuing problems [14]. A Cochrane review conducted on the use of SMS reminders adjudged the evidence available to be low to moderate in quality. However, it did show superiority in attendance rates when compared to no reminder service or a postal reminder service [15].

Breast cancer is the most commonly occurring cancer in women and the second most common cancer overall in the world [16]. Breast cancer is the commonest cancer in the UK, accounting for 15% of all new cancer cases [17]. The importance of early diagnosis and treatment of breast cancer is paramount. Breast referral pathway takes into account the need for rapid access, so that the patients are seen by a specialist within 2 weeks of referral, usually in a rapid access new patient clinic [18]. The latter, also known as one-stop clinic, allows triple assessment at a single visit, which is currently considered as the best practice [19].

The breast unit under consideration is based at a 760-bedded District General Hospital (DGH) in the North-West of England and provides a wide range of acute services to the 352,000 population of the region [20]. The breast unit deals with approximately 3500 new breast referrals annually [21]. Reducing the number of missed and cancelled appointments would help improve revenue, hospital efficiency and ultimately patient safety [6]. There is a growing interest in making the health care services more efficient. We searched PubMed, Ovid MEDLINE®, CINAHL and Google Scholar databases using Medical Subject Headings (MESH) entry terms ‘Outpatients’, ‘Breast”, Patient appointment’, ‘Patient Non-Attendance’, ‘Telephone’, ‘Text message’, ‘Short Message Service’. Literature search found studies which looked at improving DNA in breast screening [22, 23]. However, as per the current literature, we could not find any study that attempted reducing DNAs in the breast clinics.

### Aims

The primary aim was to assess the DNA rates at rapid access new patient breast clinics and reassess the impact of contacting patients prior to clinic appointment to confirm clinic attendance and introduction of a SMS-based reminder service on DNA rates at a single breast unit in the North-West of England.

The secondary aim was to evaluate CNA rate, and any correlation between DNA and patients’ age, sex, clinic days and sessions.

## Methods

### Setting

A single breast unit at a district general hospital in the North-West of England.

### Design (Fig. 1)

The study was performed in three phases and data were collected in two cycles.

i) Phase 1/ Cycle 1- Assessment of DNA and CNA rates over a 12-month period; Retrospective collection of data.
ii) Phase 2- Implementation of changes carried out by a dedicated breast administrative team working beyond the core hours: a) patients were contacted by telephone prior to offering a clinic appointment to confirm their attendance; b) letters were sent as an additional measure of confirmation of the appointment, and c) a SMS-based reminder was sent close to the clinic date.
iii) Phase 3/ Cycle 2- Re-assessment of DNA and CNA rates; Prospective collection of data.

**Fig. 1:**
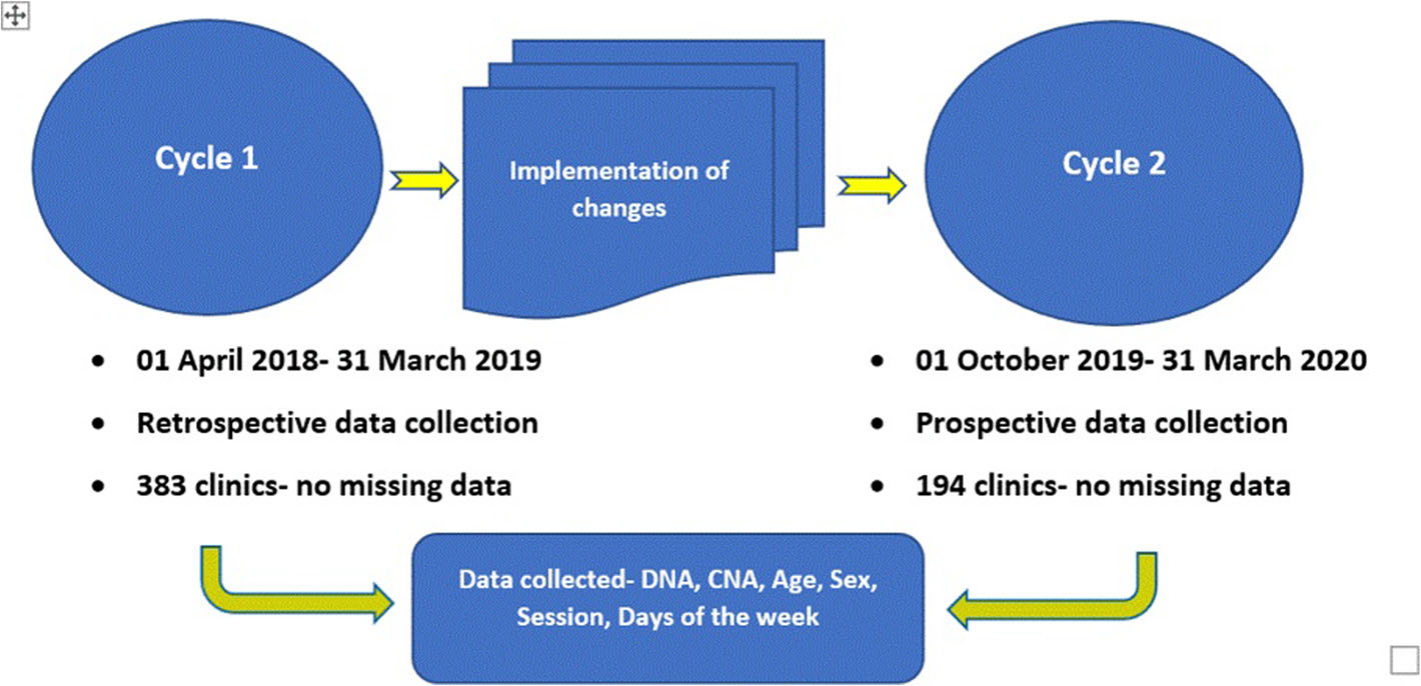
Flow diagram of study design and data collection (DNA- Did Not Attend; CNA- Could Not Attend)

### DNA and CNA rates were calculated as follows [12]

i) DNA rate= $$ \frac{\mathrm{Number}\ \mathrm{of}\ \mathrm{missed}\ \mathrm{appointments}\times 100}{\mathrm{Total}\ \mathrm{attendances}+\mathrm{Number}\ \mathrm{of}\ \mathrm{missed}\ \mathrm{appointments}} $$
ii) CNA rate= $\frac{\mathrm{Number}\ \mathrm{of}\ \mathrm{cancelled}\ \mathrm{appointments}\times 100}{\mathrm{Total}\ \mathrm{attendances}+\mathrm{Number}\ \mathrm{of}\ \mathrm{cancelled}\ \mathrm{appointments}}$

### Time line of the study period was as follows

i) Phase 1 and Cycle 1- Between 01 April 2018 and 31 March 2019
ii) Phase 2- Between April 2019 and September 2019
iii) Phase 3 and Cycle 2- Between 01 October 2019 and 31 March 2020

### Inclusion criteria

All patients seen, or expected to be seen in the rapid access new patient breast clinic during the study period.

### Exclusion criteria

None.

### Approval

The study was approved and supported by the hospital Research and Development (R & D) committee as a service improvement project, confirming that National Research Ethical approval is not required. Informed patient consent was not required in view of the fact that this was a service development project without any patient participation entailing additional clinical intervention; or sharing of identifiable individual information.

### Data collection included following information

i) Number of patients- a) expected to attend; b) cancelled appointments in advance; c) attended the clinic, and d) failed to attend without prior intimation.
ii) Clinic sessions- a) morning b) afternoon; c) evening.
iii) Clinic days- a) weekdays and b) weekends.
iv) Anonymised patient demographics- a) age and b) sex of patients who did not attend.

### Statistical analysis

Statistical analysis was performed using SPSS version 25 (IBM® Corp; Armonk, NY), entailed following tests and statistical significance was taken to be *p* < 0.05-

i) Chi-Square test (used for testing relationships on categorical variables and to compare observed results with expected results, such as sex distribution of patients)
ii) Fisher’s Exact test (applied for analysis of contingency tables, when sample sizes were small and expected values were ≤ 5, such as very low incidence of attendance)
iii) Student’s t-test (used to determine any difference between two sets of means, such as age group).

## Results

A total of 3600 new patients were expected to attend 383 rapid access breast clinics between 01 April 2018 and 31 March 2019 (Cycle 1). During this 12-month period of Phase 1 (prior to implementation of changes), 33 patients cancelled their appointments in advance and 293 patients did not attend the clinic with a median age of 38 years, (DNA rate = 8.2%). Following implementation of changes (Phase 3), which entailed establishing contacts successfully with all patients by phone prior to sending appointments, a total of 1782 new patients were expected to attend 194 clinics between 01 October 2019 and 31 March 2020 (Cycle 2). Twenty-one patients cancelled their appointments in advance in Cycle 2 and 73 patients did not attend clinic appointments with median age of 47 years, (DNA rate = 4.1%). The reduction in DNA rates was found to be statistically significant, except amongst male DNAs (Table 1). Patients who did not attend clinics in cycle 2 were significantly older than those in cycle 1 (Table 1).

**Table 1:** Distribution of attendances, DNA, age and CNA according to the intervention

	Pre-intervention (Cycle 1)	Post-intervention (Cycle 2)	*p*-value
Attended clinic (total)	3274	1688	**< 0.00001**
DNA (total)	293	73	
Attended clinic (Male)	208	110	0.906
DNA (Male)	14	7	
Attended clinic (Female)	3066	1578	**< 0.00001**
DNA (Female)	279	66	
Age (DNA) (total)	Median 38 (range, 14–85)	Median 47 (range, 15–92)	**0.0004**
Expected (total)	3600	1782	0.369
CNA (total)	33	21	

*DNA* Did Not Attend, *CNA* Could Not Attend

Sub-group analysis comparing DNA rates of weekdays with weekend showed that most of the clinics took place during the weekdays. Overall (both cycles combined) DNA rates of clinics during the weekdays and weekends were 6.9 and 4.7%, respectively (*p* = 0.28). Intervention made a statistically significant difference in total and female DNA rates during the weekdays (*p* < 0.00001), but not weekends (*p* = 0.14). DNA age group in post-intervention phase was significantly older than pre-intervention phase (0.0002) during weekdays, but no such association was noted during the weekends (Table 2).

**Table 2:** Occurrences of DNA as per weekdays and weekends

	Pre-intervention (Cycle 1)	Post-intervention (Cycle 2)	*p*-value
Weekdays			
Attended clinic (total)	3230	1573	**< 0.00001**
DNA (total)	289	69	
Weekends			
Attended clinic (total)	27	132	0.140
DNA (total)	3	5	
Weekdays			
Attended clinic (Male)	206	103	1.000
DNA (Male)	14	7	
Weekends			
Attended clinic (Male)	2	7	NA
DNA (Male)	0	0	
Weekdays			
Attended clinic (Female)	3024	1470	**< 0.00001**
DNA (Female)	275	62	
Weekends			
Attended clinic (Female)	25	125	0.132
DNA (Female)	3	5	
Weekdays			
Age (DNA) (total)	Median 38 (Range, 14–85)	Median 47 (Range, 15–92)	**0.0002**
Weekends			
Age (DNA) (total)	Median 36 (Range, 28–38)	Median 40 (Range, 23–54)	0.606

Overall DNA rates in the morning, afternoon and evening clinics were 7.7, 6.5 and 5.3%, respectively (*p* = 0.07). Significant reductions in the occurrence of DNA in the morning (*p* = 0.0001) and afternoon (*p* < 0.00001) clinics were noted following intervention. The latter did not make any significant difference in the DNA rate of evening clinics. However, the patients who did not attend the evening clinics in post-intervention phase were significantly older than pre-intervention group (Table 3).

**Table 3:** Total attendance, DNA and age distribution as per clinic session

	Pre-intervention (Cycle 1)	Post-intervention (Cycle 2)	*p*-value
Morning			
Attended clinic	1249	732	**0.0001**
DNA	129	37	
Afternoon			
Attended clinic	1527	854	**< 0.00001**
DNA	135	31	
Evening			
Attended clinic	498	102	0.728
DNA	29	5	
Morning			
Age (DNA)	Median 38 (Range, 14–85)	Median 47 (Range, 19–92)	0.009
Afternoon			
Age (DNA)	Median 40 (Range, 14–73)	Median 40 (Range, 15–83)	0.251
Evening			
Age (DNA)	Median 29 (Range, 16–65)	Median 52 (Range, 46–65)	**0.002**

The incidence of DNA was not significantly higher amongst the male (*p* = 0.61). The odds ratio for male sex as a risk factor for DNA was 1.12 (Table 4).

**Table 4:** Distribution of clinic attendance and DNA as per sex

	Attended clinic	DNA	OR (95% CI)
Male	318	21	1.12 (0.71–1.77)
Female	4644	345	

*OR* Odds Ratio, *CI* Confidence Interval

Table 5 shows highest occurrence of male as well as female DNAs on Mondays and weekdays, Least DNAs were noted in the evening clinics amongst both male and female patients.

**Table 5:** Incidence of DNA as per sex, tabulated according to sessions, weekdays, and weekends, as well as days of the week

	Male DNA	Female DNA	*p*-value
Morning	5	161	0.12
Afternoon	13	153	
Evening	3	31	
Weekday	21	337	0.48
Weekend	0	8	
Monday	8	135	0.55
Tuesday	6	89	
Wednesday	5	88	
Thursday	1	23	
Friday	1	2	
Saturday	0	5	
Sunday	0	3	

The CNA rate over a 12-month period prior to the intervention (Cycle 1) was 0.9%. Post-intervention (Cycle 2) CNA rate 1.1%. There was no significant difference in CNA rates between Cycle 1 and Cycle 2 (*p* = 0.36) (Table 1). All the clinic cancellations took place amongst the female patients only (male CNA rate = 0%). Slightly higher proportion of clinics were cancelled over the weekends. Most of the clinic cancellations took place on Mondays and in the afternoon (Table 6).

**Table 6:** Occurrence of all cancellations in both cycles combined, as per sessions and days of the week

	Number of appointments scheduled originally	Number of Cancellations	Total CNA rate (%)
Monday	1632	26	1.6
Tuesday	1438	9	0.6
Wednesday	1549	12	0.8
Thursday	574	5	0.9
Friday	20	0	0.0
Saturday	90	1	1.1
Sunday	79	1	1.3
Morning	2163	16	0.7
Afternoon	2581	34	1.3
Evening	638	4	0.6
Weekday	5213	52	1.0
Weekend	169	2	1.2

## Discussion

‘Did Not Attend’ (DNA) has impact on resources and outcomes [1]. Unfortunately, DNA remains a global issue [2]. The financial cost of annual DNAs in NHS England is equivalent to staggeringly high 257,000 hip replacements or 990,000 cataract operations [10].

Incidence of breast cancer in the UK is approximately 55,200 per annum, and is projected to rise by 2% between 2014 and 2035 [16]. Cancer Waiting Times standards monitor the length of time that patients with cancer or suspected cancer wait to be seen and treated in England [24]. These were first introduced through the NHS Cancer Plan (September 2000). The current measures and the operational standards include:

1. Two weeks from urgent GP referral for suspected cancer to first outpatient attendance (93% target)
2. Two weeks from referral with breast symptoms (where cancer is not suspected) to first hospital assessment (93% target).

All new breast referrals are seen at the rapid access breast clinic, which allows Triple assessment in a single visit, hence the clinic is also known as one-stop breast clinic. Triple assessment includes clinical assessment, radiological evaluation (mammogram and/ or ultrasound scan) and / or tissue sampling (biopsy or cytology). Needless to say, triple assessment involves in-depth multidisciplinary input and is time-consuming. Patients are made aware in advance of the waiting time for triple assessment, which can sometimes take up to 4 h. Given the nature of the assessment, only a limited number of patients can be seen in a rapid assessment breast clinic. In our set-up, the clinic template provides slots for ten new patients per clinician per session. Clinics are held usually within routine working hours and during weekdays. Depending on the workload, sometimes clinics are held during weekends and evenings as well. Waiting times are under constant scrutiny and NHS Foundation Trusts are held accountable through Monitor via the NHS Foundation Trust (NHSFT) Compliance Framework [25]. Any DNA, therefore, can lead to an increase in the waiting time, be costly, and reduce productivity [26]. It is expected that organisations should be monitoring DNA data and making a decision locally on what is an acceptable DNA rate for the organisation [25].

During first phase of our study, we initially assessed the DNA rate over a 12-month period in retrospect (Cycle 1), which was found to be 8.2%. The Trust all-specialty DNA rate for the same period was 8.8%. Our findings are in accordance with annual average 2018/2019 DNA rates of 8.6 and 7.8% noted across the NHS in England and Northern Ireland [12, 27], respectively. There is no NHS data available for comparison of DNA in the breast clinic. NHS Northern Ireland data provides an insight into DNA rates of different specialties. For example, the highest DNA rate in Northern Ireland NHS during 2018/19 was Urology, with a rate of 14.6%, followed by Dermatology (9.3%), Cardiology (9.2%), Trauma and Orthopaedic Surgery (8.7%), ENT (8.3%) and Ophthalmology (5.1%). Most of the DNAs in our study took place during weekdays and specifically on Mondays. Least DNAs were noted in the evening clinics. These might reflect rigid commitments at work or home.

In some cases, DNAs may be associated with clinical risk or less favourable outcomes, for which hospitals may face financial and regulatory penalties [28]. Providers should therefore ensure there are local policies in place to deal with DNAs and clinic cancellations by the patients (CNAs), which would reflect the spirit of cancer access guidance [25]. NHS Scotland has issued guidelines about managing DNAs [29]. NHS Improvement has provided tools for reducing DNAs [26]. Many Trusts have adopted guidelines and put local DNA policies in place [28, 30]. Communication failure, short notification, timing or day of appointment, age and sex, have all been cited as important reasons for DNA [26]. We therefore considered these factors in our study.

Changes were implemented during second phase of the study in order to address potential causes of DNA. Hospitals, as per the Department of Health (DoH) guidelines, aim to give appointments to rapid access new patient breast clinics within 2 weeks of referral in order to maintain a minimum target of 93%. Therefore, quite often, patients are offered appointments at a short notice. It is a traditional practice to send the clinic appointment letters by post. Unfortunately, letters don’t always get delivered on time. Also, the appointment date and timing may not always be suitable to the patients. Therefore, a dedicated breast appointment team was designated to work beyond routine hours and make contacts with patients by phone prior to offering appointments. This served two purposes, namely it mitigated uncertainty over postal delays and patients could opt for the days and timings most convenient to them. Subsequently, appointment letters were sent by post as well. To be sure, a SMS reminder was also sent close to the clinic date. These steps helped address potential underlying causes of DNA, such as poor communication, short notification, and inconvenient timing or day of appointment [26]. Prior to embarking on data collection (cycle 2), we allowed a period of 6 months for implementing the changes. The six-month period was deemed necessary and adequate to sort out any teething issues encountered during the process. The change in the practice continued through the whole of cycle 2.

Third phase of the study, which involved assessment of DNA following implementation of changes, showed a DNA rate of 4.1% (Cycle 2), a significant improvement by 50%, compared to Cycle 1. This is not surprising as patients found it convenient to be contacted beforehand and could make necessary adjustments at work and/ or home. It is worth emphasizing that the reduction in DNA rate was achieved despite raised DNAs that took place at the end of March 2020 due to COVID-19 pandemic, which affected every aspect of the National Health Service. Some studies have shown a reduction in missed clinic appointments, to a varied degree, following interventions [6, 13, 15]. However, no study has been performed involving direct patient contact prior to appointment and SMS alert nearer the time in regards to breast clinics. There is still a paucity of well-conducted SMS alert-based studies in reducing DNA [31]. Unfortunately, in some cases in our study, it was not possible to send SMS alerts, particularly if the patients kept the mobiles switched off or simply did not have any mobile phones. SMS alerts rely on mobile phone technology. It’s true that not growing up with technology from a young age can put older generations at a disadvantage to start learning, and that age-related health issues can make navigating a smartphone much trickier [32]. We therefore looked at age distribution of those who did not attend.

The median ages of patients who did not attend appointments following intervention were almost 9 years older, compared to the pre-intervention group. One explanation for this may be that by providing a mobile phone-based reminder service we are catering more for the younger population and this may not be suitable to reduce the number of DNA in the older population, who are more likely to be reluctant in using mobile phone technology. One corollary that consequently follows is that breast cancer, which predominantly affects the older population, may potential be missed as a result [33].

An analysis of outpatient appointment DNA data in the NHS Highland found the risk of DNA to be higher for men than women [34]. Hence, we explored the possibility that sex could be a risk factor and hypothesised male sex as a potential for higher DNA. However, the odds ratio (1.12) ruled out any such association between sex and breast clinic attendance.

Some have compared DNA with the ‘No Show’ encountered in the airline practice, which sometimes overbooks seats in anticipation of no-shows [35]. A similar action entailing overbooked clinics have been tried. But unlike airlines, clinics cannot refuse (or ‘bump’ as colloquially known in the airline practice) patients from being seen, Unsafe practice may ensue and serious capacity issue can occur, if all patients turn up on the day [35]. Therefore, blind overbooking of clinics simply is not a solution [36]. Instead, the underlying booking processes should be optimized. This would explain, rather than overbooking the clinics, why we endeavored to make changes in our booking process to address the issue with DNA,

Sometimes patients cancel in advance (CNA), even at a short notice, which can change the official DNA rate [37]. Due to late cancellation of the clinics, the vacant slots may not always be taken by other patients [1]. We therefore assessed CNA rate as well, which were 0.9 and 1.1% in Cycle 1 and Cycle 2, respectively. There was no statistically significant difference in the occurrences of CNA between Cycle 1 and Cycle 2. Lack of any significant change between pre- and post- intervention CNA rates could be explained by the fact that the occurrence of CNA was very low (0.9%) to start with. A larger study over a longer period would be warranted to assess whether, despite a low baseline rate, it is possible to reduce CNA rate significantly by pre-emptive actions. Out of four countries in the UK, NHS Northern Ireland is the only NHS body that has published data on CNA. In fact, the CNA figures in our study are lot better than the available data from the NHS Northern Ireland that showed a CNA rate of 11.3% for 2018/2019, which remained mostly unchanged compared to preceding years [12]. The speciality with the highest CNA rate in Northern Ireland was Chemical Pathology (20.4%). Once again, like DNA, no data was available on CNA for breast clinics. However, a relatively low CNA rate, as noted in our study, is a welcoming finding. Interestingly, we also noted that most of the cancellations of clinics took place on Mondays and in the afternoon. This perhaps reflects unexpected changes or situations that patients may sometimes face, which are unavoidable and can’t be swayed by SMS alerts. Awareness of higher rate of cancellation on Mondays or of afternoon sessions helped our appointment team stay alert, so that vacant slots could be offered to other patients. NHS Scotland has issued guideline as how to define and manage CNAs, assuming a reasonable offer of appointment has been made [29]. Late cancellation can interfere with the ability to utilize clinic capacity fully and some Trusts feel that insufficient notice of cancellation should be classed as DNA rather than a CNA. However, such a premise potentially introduces a subjective element to the criteria of ‘insufficient’ timescales deemed appropriate by different Trusts. Hence, as per NHS Scotland, the current definition of CNA remains unchanged [38]. However, NHS Wales guidance states that ‘When a patient contacts the Trust to cancel a second appointment, the Trust may treat the cancellation as a DNA and not make an appointment’ [39].

DNA rates have been noted to decline monotonically over the week, as found by Ellis et al. [40]. We therefore assessed the association between DNA and days of the week. Highest occurrence of DNAs took place on Mondays, least DNAs were noted in the evening clinics, which was not significantly affected by the intervention. The reasons for this may be because evening (being after office hours) is the most accessible time for the majority of the working population, requiring minimum adjustments at work place. Evening clinics also allow working partners of patients the flexibility to cater for the child-care. Hence, evening clinics can be considered as a model for those set-ups with a high DNA rate. By virtue of being aware of the distribution of DNA amongst weekdays and sessions, it is possible to reduce DNA rates by modifying appointment allocation strategy [40].

Cost of each DNA in NHS England in 2017/2018 was assessed at £120 [10]. Our study showed that even prior to the intervention, the breast unit was performing slightly better than national average in terms of clinic DNA rates [8, 9]. With the application of our intervention, we were able to reduce 73 projected DNAs over 6 months. The latter equated to a projected £17,520 annual savings due to missed rapid access new patient breast clinic appointments, not taking into account the potential penalties for unachieved targets as well as extra health and financial implications of possible missed cancers through DNAs. The actual financial saving would be more than above figure as the cost of referral to one-stop (rapid access) new patient breast clinic is significantly higher than general clinic in view of the extra time and resources required for triple assessment. However, our Trust currently holds a block contract for the breast services with the Clinical commissioning Group (CCG) and therefore, we could not confirm the individual cost for the breast clinic referral on its own. The annual efficiency saving would help fund the appointment team working beyond core hours and could potentially make the project self-sustainable.

In summary, it is possible to significantly reduce DNAs in new patient rapid access breast clinics by introducing changes such as contacting patients prior to giving appointments and sending SMS alerts. One has to be mindful of the limitation of mobile phone technology that can potentially disadvantage the older age group. The study also showed a very low CNA rate, and that patient’s sex was not a risk factor for DNA in breast clinic. Evening sessions encountered least DNAs and opens the possibility of holding evening sessions as an option to reduce DNAs. Therefore, it would be worth a consideration whether such changes in practice (namely, contacting patients during extended hours prior to offering appointments and sending SMS alerts close to clinic dates) could be implemented across the board in order to attempt to reduce DNA rates.

Limitations of our study include a relatively smaller number of male patients, shorter period of study in the post-intervention period and the focus on a speciality clinic. General clinics may have different set-ups and our experience may not necessarily be transferrable to all set-ups. Nevertheless, the changes introduced in our study are essentially a reflection of good practice and can still be used as a model for introducing changes. Moreover, this is the first study on DNA involving a speciality breast clinic and our findings will add to the existing literature in addressing reduction of DNAs.

## Conclusions

Contacting patients prior to clinic appointments and sending Short Service Message reminders nearer the clinic dates help in reducing DNA rates significantly in rapid access new patient breast clinics. Scheduling clinics on certain times and days with least DNA rates, should be contemplated. This model could be considered across the board to improve the rate of clinic DNA, that remains a global issue.

## Data Availability

The data that support the findings of this study are available from the Blackpool Teaching Hospitals NHS Foundation Trust, but restrictions apply to the availability of these data, which were used under license for the current study, and so are not publicly available. Data are however available from the authors upon reasonable request and with permission of the Blackpool Teaching Hospitals NHS Foundation Trust.

## Abbreviations

DGH: District General Hospital
DNA: Did Not Attend
CNA: Could Not Attend
SMS: Short Message Service
NHS: National Health Service
R&D: Research and Development
NHSFT: National Health Service Foundation Trust
DoH: Department of Health
CCG: Clinical Commissioning Group
